# Optically tunable terahertz chiral metasurface based on multi-layered graphene

**DOI:** 10.1038/s41598-020-60097-0

**Published:** 2020-02-21

**Authors:** Maxim Masyukov, Anna Vozianova, Alexander Grebenchukov, Kseniya Gubaidullina, Anton Zaitsev, Mikhail Khodzitsky

**Affiliations:** 1 Terahertz Biomedicine Laboratory, ITMO University, St. Petersburg, 199034 Russia; 2 International Scientific and Research Institute of Bioengineering, ITMO University, St. Petersburg, 197101 Russia

**Keywords:** Graphene, Metamaterials, Terahertz optics

## Abstract

Active manipulation of the polarization states at terahertz frequencies is crucially helpful for polarization-sensitive spectroscopy, having significant applications such as non-contact Hall measurements, vibrational circular dichroism measurements and anisotropy imaging. The weakness of polarization manipulation provided by natural materials can be overcomed by chiral metamaterials. Chiral metamaterials have a huge potential to achieve the necessary polarization effects, hence they provide the basis for applications such as ultracompact polarization components. Terahertz chiral metamaterials that allow dynamic polarization modulation of terahertz waves are of great practical interest and still challenging. Here, we show that terahertz metasurface based on the four conjugated “petal” resonators integrated with multi-layered graphene (MLG) can enable dynamically tunable chiroptical response using optical pumping. In particular, a change of ellipticity angle of 20° is observed around 0.76 THz under optical pumping by a 980 nm continuous wave (CW) laser. Furthermore, using temporal coupled-mode theory, our study also reveals that the chiroptical response of the proposed multi-layered graphene-based metasurface is strongly dependent on the influence of optical pumping on the loss parameters of resonance modes, leading to actively controllable polarization states of the transmitted terahertz waves. The present work paves the way for the realization of fundamental terahertz components capable for active polarization manipulation.

## Introduction

The polarization state is an important characteristic of light, accordingly converting and manipulating the polarization of light are crucial for many potential photonics applications. A great number of polarizers^1^ and polarization components for spectroscopy exists, such as waveplates^2^, polarization converters^3–5^, polarization-sensitive absorbers^6–9^, polarization rotators^10^, etc^11^. Metamaterials are convenient to design polarizers, particularly in terahertz frequency range, where the majority of bulk media are transparent. However, the large absorption and strong dispersion associated with the resonant response of metal structures, as well as difficulties in the manufacture of micro- and nanostructured three-dimensional objects, complicate the practical application of such media. An alternative to complex three-dimensional structures can be flat metamaterials with a thickness tens of wavelength, called metasurfaces. They consist of a single-layer^12^ or multi-layer^13^ stacks of flat structures that can be easily made by nanoprinting and lithography techniques, and the extremely thin thickness in the direction of wave propagation can significantly suppress undesirable losses. Planar metamaterials, or metasurfaces^14^, provide a spatially varying optical response and provide the simpler integration of functional materials to achieve active control of transmitted or reflected light parameters (intensity, phase, polarization, etc.) and significant amplification of nonlinear optical response^15–17^. Of particular interest are chiral metasurfaces as a tool to change the polarization state of light, due to their abilities of circular dichroism^18^, optical activity^19–21^ and negative refraction^22,23^. Usage of functional materials (e.g. carbon nanotubes, vanadium dioxide, molybdenum disulfide, graphene and its modifications) which can change their properties under external influence, such as optical pumping (OP), where the chiral properties switch is based on the metamaterial substrate photoexcitation^24–26^, heating^27,28^, impact of magnetic^29–31^ or electric field^5,32–38^ permits to design a tunable polarization converter. Existing active meta-devices with thermal tuning mechanisms consist of complementary electric split-ring^27^ or cross-shaped^28^ resonators embedded with vanadium dioxide, a phase change metasurface. Vanadium dioxide (VO_2_) exhibits a reversible phase transition at low temperatures which is critical for realizing low-power devices. Likewise, further tunable material used in the creation of terahertz and optical components for wave properties manipulation is molybdenum sulfide (MoS_2_), which has several applications in development of waveguides^39^, functional metasurfaces^40^, etc. In this case, tunable properties may be achieved by using external CW optical pumping^41^. Another promising polarization manipulation material with an extraordinary electrical and optical properties is graphene that has attracted great research interest in terahertz spectroscopy applications such as polarization rotation. The electric and optical properties of graphene (defined by Fermi energy of charge carriers) can be efficiently controlled by optical pumping^42^ with certain intensity in visible and infrared range of wavelengths, and/or by magnetic field^43^ which can be generated by neodymium magnets, and also by temperature. In addition to optical pumping, graphene Fermi energy can be rapidly changed via gate voltage. This feature makes graphene a promising material for reconfigurable devices, such as meta-deflectors^44^, meta-lenses^45^ etc. in the infrared, terahertz and microwave regions^46–48^. Thus, the integration of graphene in metasurfaces allows the creation of universal devices for polarization states control in terahertz frequency range. Due to the fact that graphene monolayer weakly interacts with pumping infrared radiation, we decided to use the multi-layered graphene in order to get rid of this drawback and to increase the efficiency of THz wave polarization control.

In this work, we firstly propose a MLG-based chiral metasurface with tunable transmission and polarization properties under the impact of optical pumping. The results of numerical simulation were considered in terms of temporal coupled-mode theory. The tunability based on the optical pumping (OP) provides the wide change of ellipticity angle about $\Delta \eta$ = 20° at the frequency of ν = 0.76 THz.

## Results

### Numerical simulation

**Figure 1:**
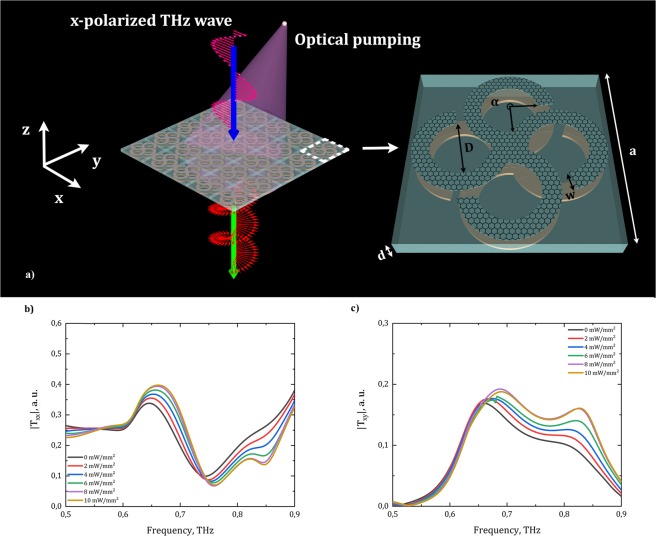
**(a)** The scheme of polarization conversion provided by the proposed metasurface and the unit cell geometrical parameters; **(b)** numerically calculated co-polarized transmission spectra of the metasurface under various optical pumping intensities; **(c)** numerically calculated cross-polarized transmission spectra of the metasurface under various optical pumping intensities.

The schematic representation of the polarization conversion provided by the investigated metasurface and its unit cell design are shown in Fig. 1(a). THz wave linearly-polarized along the *x*-direction normally propagates through the metasurface under the influence of optical pumping. The metasurface consists of an array of geometrically identical multi-layered graphene (MLG) chiral petal ring resonators and gold ones^49–51^, mirrored relatively to each other and placed on the top and bottom sides of the TPX substrate, respectively. The unit cell geometry were optimized to operate within the frequency range of 0.5–0.9 THz (MLG high tunability frequency range, see Methods) and are given by square lattice constant *a* = 406.5 μm. The thickness of the substrate is *d* = 80 μm, the inner diameter of the chiral resonator petal ring *D* = 120 μm, its width *w* = 38 μm, the turnaround angle is α = 265°, and the height of metallic resonator is 0.6 μm (Fig. 1(a)).

As a result of the simulation, the complex matrix of transmission ${\hat{{\boldsymbol{T}}}}_{{\boldsymbol{lin}}}$ (Jones matrix or T-matrix in a linear orthogonal base) was obtained. This matrix links the complex amplitudes of the incident and the transmitted waves in the following way^52^:

1 $$(\begin{array}{c}{E}_{x}^{t}\\ {E}_{y}^{t}\end{array})=(\begin{array}{cc}{T}_{xx} & {T}_{xy}\\ {T}_{yx} & {T}_{yy}\end{array})(\begin{array}{c}{E}_{x}^{i}\\ {E}_{y}^{i}\end{array})={\hat{{\boldsymbol{T}}}}_{{\boldsymbol{lin}}}(\begin{array}{c}{E}_{x}^{i}\\ {E}_{y}^{i}\end{array}),$$

where ${E}_{x}^{t}$, ${E}_{y}^{t}$ are complex amplitudes of the transmitted *x*- and *y*-polarized electromagnetic waves, ${\hat{{\boldsymbol{T}}}}_{{\boldsymbol{lin}}}$ is transmission matrix in a linear orthonormal base, ${E}_{x}^{i}$, ${E}_{y}^{i}$ are initial complex amplitudes of electromagnetic waves. It should be noticed that ${T}_{xx}$ and ${T}_{yy}$ describe the transmission of co-polarized electromagnetic waves, while non-zero cross-polarization coefficients ${T}_{xy}$ and ${T}_{yx}$ correspond to the conversion of linear polarization into elliptic one and/or the rotation of polarization plane.

Thus, the metasurface produces non-zero cross-polarized THz waves, which are perpendicular in comparison with the incident polarized wave. In our case, ${T}_{xx}={T}_{yy}$ and ${T}_{xy}=-\,{T}_{yx}$ due to the four-fold symmetry of the structure. The transmission spectra $|{T}_{xx}|$ and $|{T}_{xy}|$ are shown in Fig. 1(b,c).

The increasing power of optical pumping leads to the increment of amplitude for cross-polarized waves at the frequencies higher than 0.65 THz, while there is almost no effect on amplitudes below this frequency. One can see that the spectrum $|{T}_{xy}|$ (Fig. 1(c)) can be represented as a sum of two Lorentz-like functions which correspond to resonances in the bottom (metallic) and top (MLG) resonators with central frequencies of ν_1_ = 0.65 THz and ν_2_ = 0.83 THz, respectively. The second peak may be associated with processes in MLG resonator due to the Q-factor variation, which depends on the OP intensity.

### Transmission of circular polarization and theoretical analysis

To proceed the further analysis of the metasurface polarization conversion properties, the transmission spectra for right-handed circularly-polarized (${T}_{++}$) and left-handed circularly-polarized (${T}_{--}$) waves were calculated by the change of the base from linear orthogonal one (${\hat{{\boldsymbol{T}}}}_{{\boldsymbol{lin}}}$) to the circular one ${\hat{{\boldsymbol{T}}}}_{{\boldsymbol{cir}}}$ using the following formula:^53^

2 $${\hat{{\boldsymbol{T}}}}_{{\boldsymbol{cir}}}=(\begin{array}{cc}{T}_{++} & {T}_{+-}\\ {T}_{-+} & {T}_{--}\end{array})=\frac{1}{2}(\begin{array}{cc}{T}_{xx}+{T}_{yy}+i({T}_{xy}-{T}_{yx}) & {T}_{xx}-{T}_{yy}-i({T}_{xy}+{T}_{yx})\\ {T}_{xx}-{T}_{yy}+i({T}_{xy}+{T}_{yx}) & {T}_{xx}+{T}_{yy}-i({T}_{xy}-{T}_{yx})\end{array}),$$

where ${T}_{++}$ and ${T}_{--}$ are the transmission coefficients for right-handed and left-handed circularly polarized waves respectively, ${T}_{+-}$ and ${T}_{-+}$ describe circular cross-polarization conversion.

In general, all the complex components of transmission matrix are different if a structure does not have any rotational symmetry. In case of the chiral resonator, due to the C_4_ rotational symmetry, the transmission matrix ${\hat{{\boldsymbol{T}}}}_{{\boldsymbol{cir}}}$ is simplified into the following formula:

3 $${\hat{{\boldsymbol{T}}}}_{{\boldsymbol{cir}}}=(\begin{array}{cc}{T}_{++} & {T}_{+-}\\ {T}_{-+} & {T}_{--}\end{array})=(\begin{array}{cc}{T}_{xx}+i{T}_{xy} & 0\\ 0 & {T}_{xx}-i{T}_{xy}\end{array}),$$

Thus, the transmission coefficients for right-handed circularly-polarized (RCP) and left-handed circularly-polarized (LCP) waves are:

4 $${T}_{\pm \pm }={T}_{xx}\pm i{T}_{xy},$$

**Figure 2:**
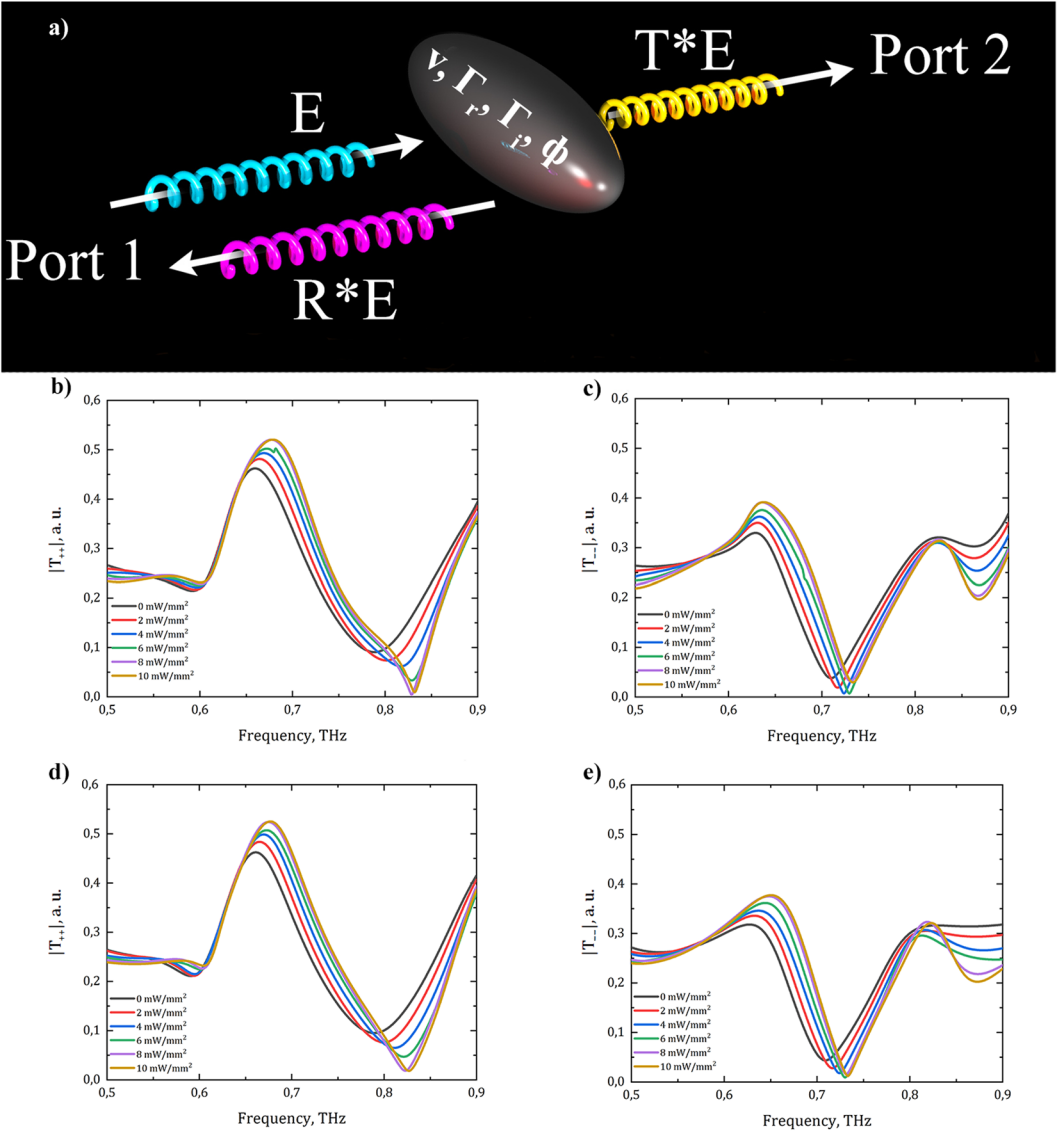
**(a)** Temporal coupled-mode theory representation: E_0_ is the amplitude of the incident THz wave, R and T are transmission and reflection coefficients, respectively, ν, ${\Gamma }_{r}$, ${\Gamma }_{i}$, $\varphi$ are the parameters of temporal coupled-mode theory approximation; **(b)** transmission of RCP wave through the metasurface obtained by numerical simulation; **(c)** transmission of LCP wave through the metasurface obtained by numerical simulation; **(d)** transmission of RCP wave through the metasurface fitted by TCMT; **(e)** transmission of LCP wave through the metasurface fitted by TCMT.

To analytically describe the obtained transmission spectra for circularly-polarized waves, one can apply temporal coupled-mode theory (TCMT)^54^. The schematic view of the model is shown in Fig. 2(a).

Due to the uncoupled nature of resonances for left-handed and right-handed circularly-polarized light, this method can be applied independently to left-handed and right-handed circular polarization. Using this formalism, the contribution of each mode of resonator into the complex transmission T is the following^55^:

5 $${\rm{T}}={t}_{0}+\mathop{\sum }\limits_{n=1}^{N}\frac{{\Gamma }_{{r}_{n}}{e}^{i{\varphi }_{n}}}{i({\nu }_{n}-\nu )+{\Gamma }_{{r}_{n}}+{\Gamma }_{{i}_{n}}},$$

where ${\nu }_{n}$ denotes the resonant frequencies of the metasurface, ${\Gamma }_{{r}_{n}}$ and ${\Gamma }_{{i}_{n}}$ are the radiation and intrinsic losses of the resonances respectively, and ${\varphi }_{n}$ represents the phase delay due to the thickness of the metasurface for each resonance mode. In our case, the transmission for RCP and LCP waves can be perfectly described by the system with four resonant frequencies (N = 4). The fitted parameters can be found in Fig. 2 and Table 1. The loss parameters ${\Gamma }_{{r}_{\mathrm{1..4}}}$ and ${\Gamma }_{{i}_{\mathrm{1..4}}}$ characterise the Q-factor of each resonance.

**Table 1:** Fitting parameters for RCP and LCP waves resonant frequencies used in TCMT.

OP, mW/mm^2^	ν_1RCP_, THz	ν_2RCP_, THz	ν_3RCP_, THz	ν_1LCP_, THz	ν_2LCP_, THz	ν_3LCP_, THz
0	0.6	0.66102	0.90709	0.65387	0.80715	0.78645
2	0.6	0.66697	0.90554	0.65811	0.84032	0.79185
4	0.6	0.67193	0.90504	0.66218	0.91262	0.80546
6	0.61193	0.67637	0.90366	0.667	0.86045	0.79903
8	0.60981	0.677	0.89825	0.67293	0.91053	0.82249
10	0.61405	0.67972	0.89754	0.67538	0.89605	0.83064

**Figure 3:**
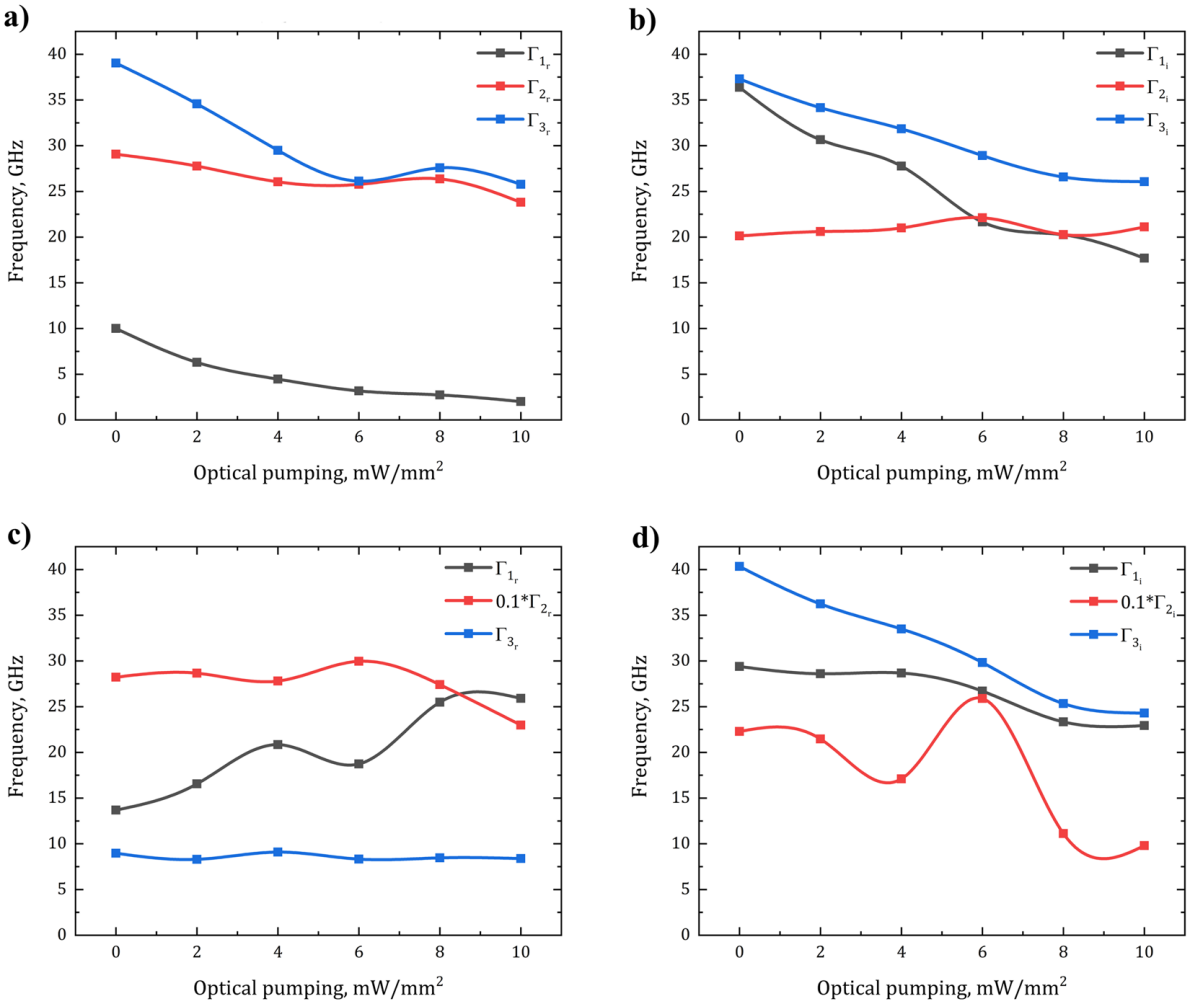
Radiation (${\Gamma }_{r}$) and intrinsic (${\Gamma }_{i}$) losses for different resonances in the metasurface depending on the optical pumping intensity for RCP **(a)**,**(b)** and LCP **(c)**,**(d)** waves, obtained by TCMT.

Figure 2(b–e) show the results of numerical simulations and analytical calculations, correspondingly, which are in good agreement. Despite of using four central frequencies for description of the transmission, only three ones give the information about the resonant processes in the frequency range of 0.5–0.9 THz. The fourth one is responsible for the total resonant process outside this frequency range (for example, transmission of RCP without optical pumping is described by ν_1..4_ = (0.6; 0.66; 0.90; 0.17) THz, where ν_4_ lays outside the discussed frequency range). The resonant frequency value dependencies on the optical pumping power values are shown in Fig. 3. It is seen that for both polarization directions there are frequency points, where the transmission is close to 0. It means that the only one polarization component passes through the structure. The optical pumping almost does not change the transmission coefficients up to 0.65 THz.

Figure 3 shows that for the RCP wave, with increasing optical pumping power, the intrinsic losses of the first and the third resonances tend to decrease, while they remain independent for the second one due to the fact that optical parameters of MLG are almost constant around this frequency. For the LCP wave, the third resonance introduces the most significant contribution to the complex transmission (at 0.84–0.90 THz). The radiation losses increase at the second resonance, while they remain almost constant for the 1^st^ and the 3^rd^ resonances. The damping rate of the second resonance is by an order of magnitude higher than for another ones, so it makes the most significant contribution to alteration of dichroic transmission.

### Polarization conversion provided by the metasurface

To analyze the polarization state transformation provided by the chiral metasurface, one should calculate the ellipticity angle of the transmitted wave. Using the data from Fig. 2, the ellipticity angle was calculated from both numerically and analytically obtained transmission spectra by the next formula^56^:

6 $$\eta =\frac{1}{2}{si}{{n}}^{-1}(\frac{{|{T}_{++}|}^{2}-{|{T}_{-}|}^{2}}{{|{T}_{++}|}^{2}+{|{T}_{-}|}^{2}}).$$

**Figure 4:**
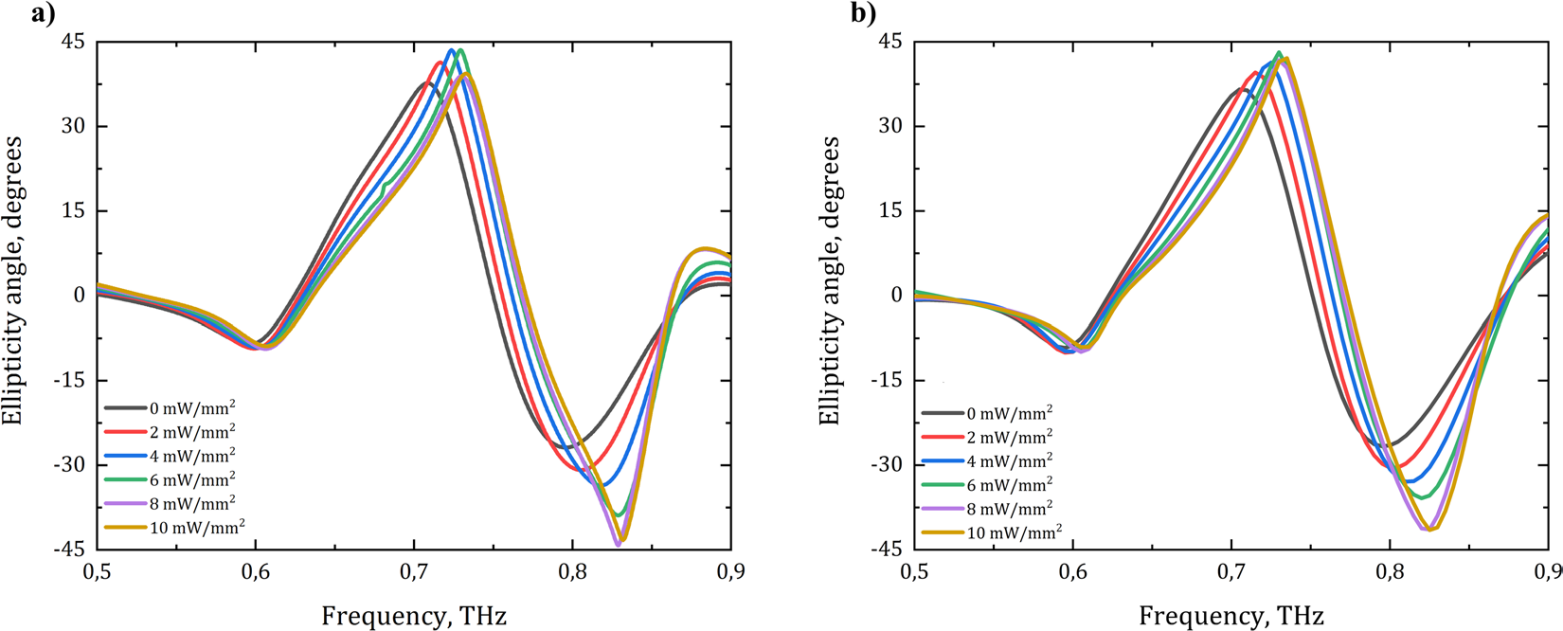
Ellipticity angle spectra of the proposed chiral metasurface depending on the value of the optical pumping obtained by **(a)** numerical simulation and **(b)** TCMT.

The value of +45° of ellipticity angle shows right-handed circularly polarized wave, −45° corresponds to left-handed circularly polarized wave, and 0° reports linearly polarized wave. The Fig. 4 demonstrates that near the frequencies of the observable circular dichroism the polarization state of the transmitted wave is transformed into circular one. The resonant frequencies of ellipticity angle shift with the increasing power of the optical pumping. The numerically calculated results fit the ones obtained by TCMT model.

**Figure 5:**
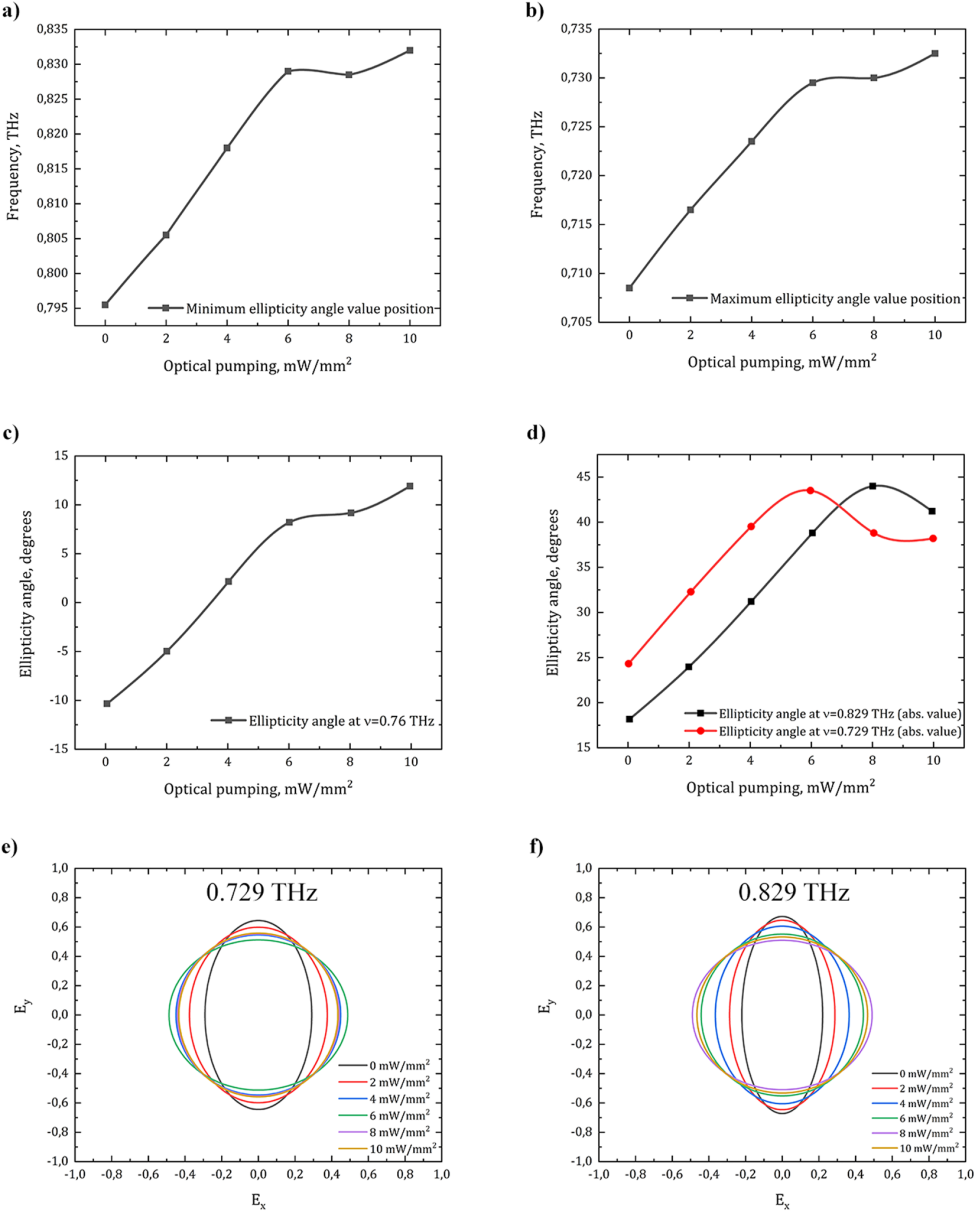
The dependence of ellipticity angle and operating frequency tunability on the power of optical pumping: **(a)** the dependence of the maximum ellipticity angle value frequency on the OP intensity; **(b)** the dependence of the minimum ellipticity angle value frequency on the OP intensity; **(c)** the conversion of polarization from left-handed to right-handed elliptically polarized one at ν = 0.76 THz; **(d)** the dependence of ellipticity angle absolute values at the frequencies of ν_1_ = 0.729 THz and ν_2_ = 0.829 THz on OP intensity; **(e)** polarization ellipse at the frequency of ν = 0.729 THz depending on the power of OP; **(f)** polarization ellipse at the frequency of ν = 0.829 THz depending on the power of OP.

To understand how the polarization changes at the certain frequencies, the absolute values of ellipticity angle |η | were calculated at frequencies of 0.729 THz and 0.829 THz (Fig. 5). It can be noticed that after reaching the maximum, the values of ellipticity angle decrease, which can be associated with saturation-like processes in MLG. Also it should be noticed that frequencies of maximal and minimal values are shifted at the value of Δν = 300 GHz by the increasing OP. The OP-based tunability shows a great value of ellipticity angle tunability, achieving $\Delta \eta$ = 20° at the frequency of ν = 0.76 THz.

## Discussion

In this article, we have proposed the polarization-selective tunable chiral metasurface. The investigated metasurface has a sandwich structure, i.e. it is composed of an array of metallic and MLG resonators placed on each side of the dielectric structure. The conductivity of the MLG resonators is controlled by optical pumping of 980 nm continuous wave laser. After the propagation through the metasurface, in general linearly-polarized incident terahertz wave is transformed into elliptically-polarized one. Transmission spectra for RCP and LCP show two different frequencies for each polarization where is no transmission can be observed, and these resonant frequencies can be tuned by the influence of OP. At these frequencies, the linearly-polarized THz wave changes its polarization into circular one. The results obtained by numerical simulations are described by temporal coupled-mode theory. TCMT shows that such mechanism is provided by the decreasing of losses in the metasurface for RCP wave and significantly high losses (in comparison with RCP waves) for LCP wave. The ellipticity angle shows a good tunability achieved by the changes of optical pumping. Because of such tunability, this metasurface can be used as double-band tunable quarter-wave plate for CW sources, narrow-band polarization filters, etc.

Device fabrication procedure includes metal and MLG films growth and its patterning. The multilayered graphene film can be produced by chemical vapor deposition (CVD) method^57^. The patterning of graphene film, as well as metal film, can be performed using UV-photolithography process^58^. To align the top (graphene) and bottom (metal) patterns the mask aligner can be used^59^.

## Methods

### Characterization of the materials used in numerical simulation

**Figure 6:**
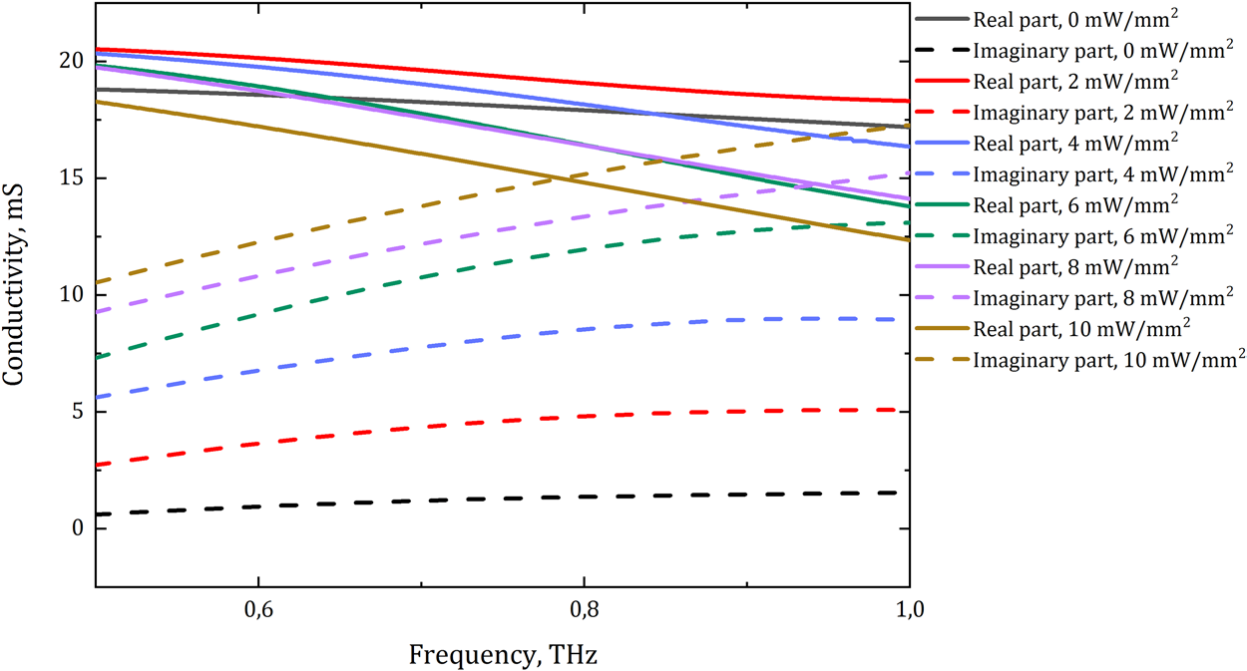
The conductivity spectra of MLG under optical pumping in THz frequency range obtained experimentally using THz-TDS measurements.

Multi-layered graphene (MLG) was fabricated using chemical vapour deposition (CVD) technique on Ni foil^42^. The nickel was chemically etched, and graphene was transferred to a polymethylpentene (TPX) substrate. In order to use the optical parameters of MLG in numerical simulations, the terahertz time-domain spectroscopy (THz-TDS) method was applied at the frequency range of 0.5–1.0 THz. The conductivity of MLG was controlled by continuous-wave optical pumping with wavelength of 980 nm. The complex conductivity spectra $\hat{\sigma }(\nu )$ of MLG (Fig. 6) for different optical pumping intensities were extracted from transmission data using the following formula^42,60,61^ in a thin-film approximation (the wavelength of THz wave is much more than the film thickness):

7 $$\hat{\sigma }(\nu )=\frac{1}{{Z}_{0}}({n}_{s}+1)(\frac{{\hat{E}}_{0}(\nu )}{\hat{E}(\nu )}-1),$$

where ${Z}_{0}$ is the impedance of free space, n_s_ is the refractive index of a substrate, ${\hat{E}}_{0}(\nu )$ and $\hat{E}(\nu )$ are complex amplitudes of THz wave transmitted through the TPX substrate and MLG on the substrate, which were obtained by Fast Fourier transform (FFT) method. The optical pumping does not affect the optical properties of dielectric TPX substrate, so it can be perfectly described by a dielectric constant ε = 2.1 in the investigated frequency range. The obtained conductivity spectra are in a good agreement with Kubo formalism and they also can be fitted using Drude-Smith formula, as it was shown in ref. ^42^.

### Numerical simulation

The numerical simulation was performed in CST Microwave Studio. A unit cell boundary conditions in the frequency domain solver were implemented. The thickness of multilayered graphene is sufficiently thin in comparison to THz radiation wavelength. Thereby, the MLG resonator was considered as infinitesimally thin impedance sheet and its parameters were simulated through tabulated surface impedance, obtained from experimental data. Thus, depending on the value of optical pumping, in each numerical simulation we used the required spectrum of MLG impedance. The substrate was the same as in the material characterization (TPX, ε = 2.1). Golden resonators properties are defined by Drude model.

### Temporal coupled-mode theory

The coupled-mode theory is based on the modes coupling in a time-dependent formalism for optical resonators. The equation of the amplitude for each resonance mode is^54^

8 $$\frac{d{a}_{n}}{dt}=(i{\omega }_{n}-{\tau }_{n}^{-1}){a}_{n}+(\langle {\kappa }_{n}|\ast )|{s}_{+}\rangle ,$$

where ${\omega }_{n}=2{{\rm{\pi }}{\rm{\nu }}}_{n}$ describes the central frequency for each resonance mode, ${{\rm{\tau }}}_{n}=2{\rm{\pi }}{({\Gamma }_{{r}_{n}}+{\Gamma }_{{i}_{n}})}^{-1}$ is the term responsible for losses, $|{s}_{+}\rangle$ represents the incoming waves and $\langle {\kappa }_{n}|$ is the coupling coefficient between the resonance mode and incoming wave. The outgoing wave $|{s}_{-}\rangle$ can be written as

9 $$|{s}_{-}\rangle =S|{s}_{+}\rangle =C|{s}_{+}\rangle +{\sum {a}_{n}}^{}|{d}_{n}\rangle ,$$

where $S$ is the scattering matrix of the whole system, $C$ is the scattering matrix which describes direct coupling between incoming and outcoming waves, $|{d}_{n}\rangle$ is the coupling coefficient between the resonance modes and outcoming waves.

In case of two ports, the ket states are the following:

10 $$|{s}_{\pm }\rangle =(\begin{array}{c}{s}_{1\pm }\\ {s}_{1\pm }\end{array}),|{d}_{n}\rangle =(\begin{array}{c}{d}_{n1}\\ {d}_{n2}\end{array}),\,|{\kappa }_{n}\rangle =(\begin{array}{c}{\kappa }_{n1}\\ {\kappa }_{n2}\end{array}),$$

The scattering matrix *S* for the whole system in case of two ports can be expressed as

11 $$S=(\begin{array}{cc}r & it\\ it & r\end{array}),$$

where $t$ and $r$ are transmission and reflection coefficients, correspondingly. The outgoing wave can be obtained using Eqs. 8 and 9 as follows:

12 $$|{s}_{-}\rangle =[C+{\sum }^{}\frac{|{d}_{n}\rangle \langle {\kappa }_{n}|\ast }{i(\omega -{\omega }_{n})+{\tau }_{n}^{-1}}]|{s}_{\pm }\rangle ,$$

The term in brackets from Eq. (9) corresponds to scattering matrix of the overall system $S$. Thus, by using Eqs. (11) and (12) from above, the transmission spectra for the system with n resonance modes can be expressed as

13 $${\rm{T}}={t}_{0}+\mathop{\sum }\limits_{n=1}^{N}\frac{1/{\tau }_{n}}{i({\omega }_{n}-\omega )+{\tau }_{n}^{-1}},$$

Assuming the presence of the intrinsic and radiation losses, phase delay and the number of resonance modes, we get formula in the main text (Eq. (5)).

## Supplementary information

Supplementary information.
