# Supplementary material for: Optically tunable terahertz chiral metasurface based on multi-layered graphene
Source: Sci Rep. 2020 Feb 21;10:3157. doi: 10.1038/s41598-020-60097-0 (PMC7035278; doi:10.1038/s41598-020-60097-0)
Supplement: Supplementary file 1 — Supplementary information. [file 41598_2020_60097_MOESM1_ESM.pdf]

# Optically tunable terahertz chiral metasurface based on multi-layered graphene - Supplementary Information


**Maxim Masyukov [1,*] , Anna Vozianova [1,2] , Alexander Grebenchukov [1,2] , Kseniya Gubaidullina [1] , Anton Zaitsev [1] , and Mikhail Khodzitsky [1,2]**

[1] Terahertz Biomedicine Laboratory, ITMO University, St. Petersburg 199034, Russia
[2] International Scientific and Research Institute of Bioengineering, ITMO University, St. Petersburg 197101, Russia
[*] msmasyukov@itmo.ru


## S1. Temporal coupled-mode theory fitted parameters for RCP and LCP waves.

**Table S1.** TCMT fit parameters obtained for right-handed circularly-polarized (RCP)

| **Pumping** | **Re($t_0$)** | **Im($t_0$)** | **$f_1$,THz** | **$f_2$,THz** | **$f_3$,THz** | **$f_4$,THz** | **$\Gamma_{r1}$,THz** | **$\Gamma_{r2}$,THz** | **$\Gamma_{r3}$,THz** | **$\Gamma_{r4}$,THz** | **$\Gamma_{i1}$,THz** | **$\Gamma_{i2}$,THz** | **$\Gamma_{i3}$,THz** | **$\Gamma_{i4}$,THz** | **$\varphi_1$** | **$\varphi_2$** | **$\varphi_3$** | **$\varphi_4$** |
|---|---|---|---|---|---|---|---|---|---|---|---|---|---|---|---|---|---|---|
| **0** | 0,1061 | -0,19616 | 0,6 | 0,66102 | 0,90709 | 0,31793 | 0,01 | 0,02907 | 0,03903 | 0,05458 | 0,03638 | 0,02012 | 0,0373 | 0,01275 | -3,4702 | -1,48704 | -1,49273 | 6,3 |
| **2** | 0,12651 | -0,1988 | 0,6 | 0,66697 | 0,90554 | 0,2985 | 0,0063 | 0,02776 | 0,03457 | 0,06856 | 0,03065 | 0,0206 | 0,03415 | 0,01805 | -3,56978 | -1,58734 | -1,38397 | 6,3 |
| **4** | 0,16509 | -0,23813 | 0,6 | 0,67193 | 0,90504 | 0,19613 | 0,00446 | 0,02604 | 0,02948 | 0,12928 | 0,02776 | 0,021 | 0,03184 | 0,02629 | -3,6105 | -1,62755 | -1,27994 | 6,3 |
| **6** | 0,18957 | -0,24501 | 0,61193 | 0,67637 | 0,90366 | 0,1784 | 0,00318 | 0,02577 | 0,02612 | 0,15535 | 0,02166 | 0,02211 | 0,02891 | 0,02807 | -4,18625 | -1,7101 | -1,15471 | 6,29833 |
| **8** | 0,19865 | -0,30266 | 0,60981 | 0,677 | 0,89825 | 0,14264 | 0,00273 | 0,02636 | 0,02757 | 0,20755 | 0,02025 | 0,02027 | 0,02658 | 0,04299 | -3,93579 | -1,65855 | -0,98552 | 6,12489 |
| **10** | 0,14361 | -0,39657 | 0,61405 | 0,67972 | 0,89754 | 0,1713 | 0,00201 | 0,02381 | 0,02577 | 0,32006 | 0,0177 | 0,02111 | 0,02606 | 0,15248 | -4,2693 | -1,69236 | -0,92419 | 5,62862 |

**Table S2.** TCMT fit parameters obtained for left-handed circularly-polarized (LCP)

| **Pumping** | **Re($t_0$)** | **Im($t_0$)** | **$f_1$,THz** | **$f_2$,THz** | **$f_3$,THz** | **$f_4$,THz** | **$\Gamma_{r1}$,THz** | **$\Gamma_{r2}$,THz** | **$\Gamma_{r3}$,THz** | **$\Gamma_{r4}$,THz** | **$\Gamma_{i1}$,THz** | **$\Gamma_{i2}$,THz** | **$\Gamma_{i3}$,THz** | **$\Gamma_{i4}$,THz** | **$\varphi_1$** | **$\varphi_2$** | **$\varphi_3$** | **$\varphi_4$** |
|---|---|---|---|---|---|---|---|---|---|---|---|---|---|---|---|---|---|---|
| **0** | 0,34024 | -0,27779 | 0,65387 | 0,80715 | 0,78645 | 0,36717 | 0,01368 | 0,28222 | 0,00897 | 0,03363 | 0,0294 | 0,22289 | 0,04034 | 0 | -2,68229 | -0,89231 | -0,52602 | -6,3 |
| **2** | 0,34617 | -0,27227 | 0,65811 | 0,84032 | 0,79185 | 0,35869 | 0,01656 | 0,28657 | 0,0083 | 0,03428 | 0,0286 | 0,21463 | 0,03624 | 0 | -2,67343 | -0,87659 | -0,74137 | -6,3 |
| **4** | 0,3764 | -0,2664 | 0,66218 | 0,91262 | 0,80546 | 0,3574 | 0,02084 | 0,27807 | 0,00909 | 0,03986 | 0,02867 | 0,17106 | 0,0335 | 0 | -2,62287 | -0,86128 | -1,27283 | -6,3 |
| **6** | 0,32163 | -0,25597 | 0,667 | 0,86045 | 0,79903 | 0,32508 | 0,01873 | 0,29971 | 0,00832 | 0,03406 | 0,0267 | 0,25887 | 0,02983 | 0 | -2,65517 | -0,84866 | -0,9259 | -6,3 |
| **8** | 0,36723 | -0,23498 | 0,67293 | 0,91053 | 0,82249 | 0,38355 | 0,02549 | 0,27409 | 0,00846 | 0,0268 | 0,02334 | 0,11123 | 0,02533 | 0 | -2,78982 | -0,83053 | -1,99846 | -6,3 |
| **10** | 0,33056 | -0,22513 | 0,67538 | 0,89605 | 0,83064 | 0,36594 | 0,0259 | 0,22985 | 0,00838 | 0,03228 | 0,02293 | 0,09802 | 0,0243 | 0 | -2,79548 | -0,82771 | -2,32855 | -6,3 |

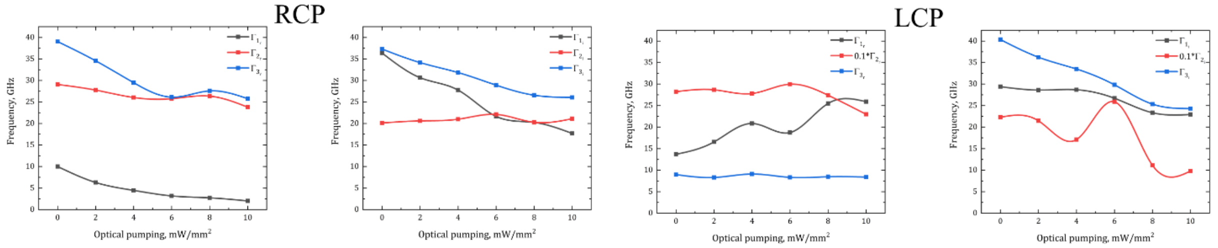


**Figure S1.** Dependence of Temporal coupled-mode theory fit parameters on intensity of optical pumping
